# A Seizure Attributed to Ofloxacine in a Woman Undergoing Detoxification for Alcohol Dependence

**DOI:** 10.1155/2009/705635

**Published:** 2010-01-24

**Authors:** Pierre Lahmek, Laurent Michel, Nadine Meunier, Henri-Jean Aubin

**Affiliations:** Centre de Traitement des Addictions, Hôpital Emile Roux, Assistance Publique-Hôpitaux de Paris, 94450 Limeil-Brévannes, France

## Abstract

*Objective*. To report one case of seizure following administration of ofloxacin. *Case Summary*. A 38-year-old woman with alcohol dependence but no prior history of seizure disorder admitted in our inpatient alcohol detoxification program was prescribed ofloxacin four days after admission for a lower urinary tract infection. She was currently prescribed diazepam 30 mg per day. This treatment was continued without modification following admission. Forty eight hours after starting ofloxacin and after receiving five doses of oral ofloxacin, the patient experienced a seizure. Ofloxacin treatment was stopped and no further seizures occurred. Neurological examination of the patient, laboratory tests, computerized tomography with contrast enhancement and electroencephalography did not detect any abnormalities. Up to the last consultation, six months after admission, the patient has reported no recurrence of the seizure. *Discussion*. Quinolone antibiotics vary in their ability to induce seizures, with ofloxacin having one of the least potentials. In the present case, the seizure could be attributed in all probability to taking ofloxacin; since she had no previous history of seizures, she did not present an alcohol withdrawal syndrome, benzodiazepine treatment was not modified, the seizure occurred 48 h after taking ofloxacin, but seven days after stopping drinking, no alternative aetiologies for the seizure could be identified and no seizure recurrence was reported over the following seven months. Of reported cases of seizures in patients treated with fluoroquinolones, none concerned patients with alcohol dependence or patients treated with benzodiazepines. *Conclusions*. The present case alerts us to the possibility that seizures may occur in alcohol dependent patients treated with benzodiazepines who concomitantly prescribed a fluoroquinolone. These widely-used antibiotics should thus be prescribed with caution to patients undergoing detoxification for alcohol dependence, particularly if they are also taking benzodiazepines, irrespective of whether they have a previous history of seizures or not.

## 1. Introduction

The fluoroquinolones are a widely-used class of broad-spectrum antibiotics, particularly useful for the treatment of urinary tract infections caused by gram-negative bacteria [1]. Effects of fluoroquinolones on the central nervous system have been reported rarely, and are non-specific. In particular, seizures are extremely rare. These have been attributed to a specific interaction of fluoroquinolones with inhibitory *γ*-aminobutyric acid (GABA) receptors on neurones, resulting in a reduction in the seizure threshold [2]. Alcohol dependence is a risk factor for seizures, in particular in individuals who present symptoms of alcohol withdrawal [3, 4]. However, to our knowledge, seizures have not so far been reported in alcohol-dependent patients treated with fluoroquinolones antibiotics whilst undergoing withdrawal. This report describes a woman with alcohol dependence but no prior history of epilepsy or seizures who experienced a seizure after taking ofloxacin during medically-supervised alcohol detoxification.

## 2. Case Report

A 38-year-old woman was admitted to our alcohol treatment centre to undergo inpatient detoxification. She presented with a history of regular high alcohol consumption since the age of 23, fulfilling diagnostic criteria for alcohol dependence (according to the DSM-IV) since the age of thirty. This was her first inpatient treatment for an alcohol-related problem. Three years prior to admission, the patient reported a previous period of abstinence following hospitalisation in a psychiatric department for the treatment of a major depressive episode. Her alcohol consumption had increased dramatically since the last four months before admission and she reported drinking between 50 and 75 cl of whisky on a daily basis (e.g., between 200 and 300 grams of ethanol per day). Her last alcohol intake was the day prior to hospitalisation and at admission her blood alcohol levels were below the threshold of detection and she presented no signs of a withdrawal syndrome according to the DSM-IV (e.g., no increasing hand tremor, transient visual tactile or auditory hallucinations, autonomic hyperactivity, insomnia, nausea or vomiting, psychomotor agitation, anxiety or grand mal seizure) at admission and during the next days. Thus no supportive measure were needed.

Her medical history included a fracture of the right ankle six years prior to admission, essential hypertension, allergic asthma, anxiety and depression. No previous history of seizures was reported. She was currently prescribed bisoprolol 10 mg per day, venlafaxine LP 75 mg *bid*, diazepam 30 mg per day, zolpidem 10 mg per day, inhaled beclometasone and salbutamol, and oral contraception with levonorgestrel. These treatments were continued without modification following admission. Clinical examination revealed varicose veins affecting both legs and minor bruising to the back and torso. No clinical signs suggestive of alcohol-related pathology were detected. At admission, clinical examination as well as routine hematology and biochemistry tests were all normal, particularly potassium and sodium levels. Magnesium level was not measured.

Four days after admission, a lower urinary tract infection was detected and *Collibacillum* identified as the pathogen responsible. For this reason, treatment with ofloxacin 200 mg *bid* was prescribed. No change was made to her other medication. Forty eight hours after starting ofloxacin, six days after admission and seven days after the last alcohol intake, the patient experienced a generalised tonic-clonic seizure lasting for five minutes followed by a post-ictal period of twenty minutes. Clinical examination of the patient in the hours following the episode only revealed a bilateral facial haematoma around the eyes as a consequence of the seizure and the fall. Neurological examination was once again normal. Ofloxacin treatment was stopped and no further seizures occurred. Laboratory tests performed the next morning were normal (blood cell count, blood chemistry, calcium, phosphorus, magnesium, blood glucose and transaminases). Computerised tomography (CT) with contrast enhancement and electroencephalography (EEG) were performed and neither detected any abnormalities. No further seizure occurred during the 21 days of hospitalisation. The patient still attends regular followup consultations at our centre. Up to the last consultation, six months after admission, she claims not to have consumed any more alcohol and has reported no recurrence of the seizure.

## 3. Discussion

This case depicts the occurrence of a generalised tonic-clonic seizure in an alcohol dependent patient without prior seizure history forty-eight hours after starting treatment with ofloxacin. Seizures unrelated to epilepsy are relatively common events, for example as complications of deregulated metabolic states (hypoglycaemia, renal insufficiency, hyperglycaemia, dehydration, hyperhydration, hepatic insufficiency, or perturbations of calcium or phosphorus homeostasis), as toxicological reactions (drug overdose or withdrawal) or following infections of the central nervous system (meningitis or encephalitis) [5, 6]. The seizures that may occur following abrupt alcohol withdrawal fall into this category of nonepileptic seizures, although they usually are not sufficiently severe to merit specific treatment or management [3, 7, 8]. Postictal neurological examination is usually normal in these patients. If withdrawal seizures occur, it is recommended to perform a basic physical and biological examination, including EEG, CT or magnetic resonance imaging, and laboratory tests appropriate to the individual situation [9–11]. After a first withdrawal seizure, anomalies can be detected on the EEG in around 50% of cases and on CT in between 35% to 55% of cases, whereas laboratory tests are usually normal [10]. However, in the absence of risk factors for repeated seizures, such as EEG anomalies or other identified seizure aetiologies, only 25% of patients will experience a second seizure in the two years following the index episode [12]. 

Excessive alcohol consumption over prolonged periods is toxic to the central nervous system and may lead to structural and functional changes. From a functional perspective, ethanol inhibits neuronal excitability by facilitating inhibitory GABAergic transmission. In alcohol dependence, tolerance develops to this facilitatory action, involving an increased sensitivity to the excitatory neurotransmitter glutamate and reduced sensitivity to GABA. When alcohol intake is abruptly ended, for example, during detoxification, this balance between excitation and inhibition is perturbed, leading to a hyperexcitable state which manifests as the alcohol withdrawal syndrome [3, 4, 7, 13, 14]. Around half of alcohol-dependent patients present a withdrawal syndrome when they stop drinking, and seizures occur in around five percent of cases. These are usually generalised tonic clonic seizures occurring in eighty percent of cases between eight hours and two days after stopping drinking [3, 4]. Benzodiazepines, notably diazepam, are the reference pharmacological treatment for managing the alcohol withdrawal syndrome [3, 4, 7, 14, 15], since they activate the GABAergic system and this attenuate the hyperexcitable state.

In the present case, the seizure could be attributed in all probability to taking ofloxacin, since she had no previous history of seizures, she did not present an alcohol withdrawal syndrome, benzodiazepine treatment was not modified, the seizure occurred 48 hours after taking ofloxacin but seven days after stopping drinking, no alternative aetiologies for the seizure could be identified and no seizure recurrence was reported over the following seven months. The possibility that the seizure was related to alcohol withdrawal seemed low, given the absence of a withdrawal syndrome or previous seizure history, and the time elapsed between stopping drinking and the seizure. It is extremely rare for alcohol withdrawal seizures to occur more than 72 hours following interruption of alcohol consumption [3, 4, 7]. Benzodiazepine withdrawal can also be excluded as the cause of the seizure, since the dose of diazepam was not changed. Following the criteria proposed by Naranjo et al. [16], it can be concluded that the seizure was probably imputable to ofloxacin. But this scale has been found to lack validity and reliability regarding causality in various situations and this would similarly be true for ofloxacin because ofloxacin induced seizures are rare. Ofloxacin may well be the cause attributable for the seizure. It is however impossible to conclude definitely on the causal relationship from this case report, considering the possible confounders, in this case alcohol detoxification.

The fluoroquinolones are a generally well-tolerated class of antibiotics. Outside the central nervous system, the adverse drug reactions most frequently reported with this treatment class are nausea and vomiting, hypo- or hyper-glycaemia, increase in the QT interval, phototoxicity, tendinopathy, and pseudomembranous colitis due to *Clostridium difficile* [17]. Adverse drug reactions involving the central nervous system include dizziness, somnolence, confusional states and tremor. The possible occurrence of seizures after taking ofloxacin is a recognised potential adverse reaction identified in the prescribing information for this drug and indeed for all fluoroquinolones antibiotics [2, 17–19]. These seizures can be attributed either to the binding of the antibiotic to the GABA_A_ receptor or to an interaction with excitatory amino acid receptors. In both cases, these interactions would lead to a reduction in seizure threshold [20]. It has been claimed that binding to the GABA_A_ receptor, and consequently seizure risk, would be enhanced by certain substitutions at the 7-position of the quinolone ring system, notably piperazine groups as in ciprofloxacin [17, 21–23]. Consistent with this, we have identified more than ten reported cases of seizures associated with other 7-piperazinyl-fluoroquinolones, such as ofloxacin, levofloxacin, enoxacin, and gatifloxacine [2, 24–33], but also with non-piperazinylated, non-fluorinated quinolones such as nalidixic acid [34, 35]. The relative convulsant potency of different fluoroquinolones has been evaluated in vitro, and trovafloxacine found to be the most potent and levofloxacin the least potent [2]. The factors that determine convulsant potency are poorly understood, but may include access to the central nervous system (which is itself related to lipophilicity), affinity for the GABA_A_ receptor and activity at excitatory amino acid receptors [1, 2, 17–19, 36–38].

When patients are treated with benzodiazepines, taking other medication like ofloxacin that may interact with the benzodiazepine-binding sites located on the same complex as the GABA receptor site could increase the risk of seizures [38]. It is possible that the case described here illustrates such a phenomenon. The seizure could have been due to displacement of GABA from its receptor, decreasing GABA-ergic inhibition [2]. In contrast to this hypothesis, Akaike et al. report that the inhibitory actions of fluoroquinolones combined with biphenyl acetic acid (the active metabolite of the NSAID fenbufen, which potentiates the inhibitory actions of fluoroquinolones) on GABA-mediated responses were not influenced by the presence of a benzodiazepine antagonist (flumazenil) indicating that their actions are probably not directly mediated via the benzodiazepine receptor [39]. Fluoroquinolone antibiotics should thus be used with precaution in patients prescribed other drugs that may also affect the seizure threshold [17]. Interactions between quinolones and certain non-steroidal anti-inflammatory drugs or theophylline may also elicit seizures through such a mechanism [20, 40, 41]. Of the seventeen reported cases of seizures in patients treated with fluoroquinolones, none concerned patients with alcohol dependence or patients treated with benzodiazepines [2, 21, 22, 24–35, 42]. In half of these cases, another risk factor for seizures could be identified, namely renal insufficiency, brain lesions, disturbances of water or electrolyte homeostasis, or drug overdose. The interval between the first administration of the fluoroquinolones and the seizure ranged between two to four days, that is between the third and eighth dose, as was also the case for the patients described here.

## References

[1] O’Donnell JA, Gelone SP (2000). Fluoroquinolones. *Infectious Disease Clinics of North America*.

[2] Kushner JM, Peckman HJ, Snyder CR (2001). Seizures associated with fluoroquinolones. *Annals of Pharmacotherapy*.

[3] Schuckit MA (2009). Alcohol-use disorders. *The Lancet*.

[4] Hughes JR (2009). Alcohol withdrawal seizures. *Epilepsy and Behavior*.

[5] Annegers JF, Dubinsky S, Coan SP, Newmark ME, Roht L (1999). The incidence of epilepsy and unprovoked seizures in multiethnic, urban health maintenance organizations. *Epilepsia*.

[6] Annegers JF, Hauser WA, Lee JR-J, Rocca WA (1995). Incidence of acute symptomatic seizures in Rochester, Minnesota, 1935–1984. *Epilepsia*.

[7] (2009). In the clinic. Alcohol use. *Annals of Internal Medicine*.

[8] Mayo-Smith MF (1997). Pharmacological management of alcohol withdrawal: a meta-analysis and evidence-based practice guideline. *Journal of the American Medical Association*.

[9] French JA, Pedley TA (2008). Clinical practice. Initial management of epilepsy. *The New England Journal of Medicine*.

[10] Harden CL, Huff JS, Schwartz TH (2007). Reassessment: neuroimaging in the emergency patient presenting with seizure (an evidence-based review): report of the therapeutics and technology assessment subcommittee of the American Academy of Neurology. *Neurology*.

[11] Krumholz A, Wiebe S, Gronseth G (2007). Practice parameter: evaluating an apparent unprovoked first seizure in adults (an evidence-based review): report of the quality standards subcommittee of the American Academy of Neurology and the American Epilepsy Society. *Neurology*.

[12] Berg AT (2008). Risk of recurrence after a first unprovoked seizure. *Epilepsia*.

[13] Krystal JH, Staley J, Mason G (2006). *γ*-aminobutyric acid type A receptors and alcoholismml: intoxication, dependence, vulnerability, and treatment. *Archives of General Psychiatry*.

[14] Sullivan JT, Sykora K, Schneiderman J, Naranjo CA, Sellers EM (1989). Assessment of alcohol withdrawal: the revised clinical institute withdrawal assessment for alcohol scale (CIWA-Ar). *British Journal of Addiction*.

[15] Sannibale C, Fucito L, O’Connor D, Curry K (2005). Process evaluation of an out-patient detoxification service. *Drug and Alcohol Review*.

[16] Naranjo CA, Busto U, Sellers EM (1981). A method for estimating the probability of adverse drug reactions. *Clinical Pharmacology and Therapeutics*.

[17] Mehlhorn AJ, Brown DA (2007). Safety concerns with fluoroquinolones. *Annals of Pharmacotherapy*.

[18] Christ W (1990). Central nervous system toxicity of quinolones: human and animal findings. *Journal of Antimicrobial Chemotherapy*.

[19] De Sarro A, De Sarro G (2001). Adverse reactions to fluoroquinolones. An overview on mechanistic aspects. *Current Medicinal Chemistry*.

[20] Akahane K, Kato M, Takayama S (1993). Involvement of inhibitory and excitatory neurotransmitters in levofloxacin- and ciprofloxacin-induced convulsions in mice. *Antimicrobial Agents and Chemotherapy*.

[21] Darwish T (2008). Ciprofloxacin-induced seizures in a healthy patient. *New Zealand Medical Journal*.

[22] Schwartz MT, Calvert JF (1990). Potential neurologic toxicity related to ciprofloxacin. *DICP, The Annals of Pharmacotherapy*.

[23] Tattevin P, Messiaen T, Pras V, Ronco P, Biour M (1998). Confusion and general seizures following ciprofloxacin administration. *Nephrology Dialysis Transplantation*.

[24] Anastasio GD, Menscer D, Little JM (1988). Norfloxacin and seizures. *Annals of Internal Medicine*.

[25] Bird SB, Orr PG, Mazzola JL, Brush DE, Boyer EW (2005). Levofloxacin-related seizure activity in a patient with Alzheimer’s disease: assessment of potential risk factors. *Journal of Clinical Psychopharmacology*.

[26] Christie MJ, Wong K, Ting RH, Tam PY, Sikaneta TG (2005). Generalized seizure and toxic epidermal necrolysis following levofloxacin exposure. *Annals of Pharmacotherapy*.

[27] Melvani S, Speed BR (2000). Alatrofloxacin-induced seizures during slow intravenous infusion. *Annals of Pharmacotherapy*.

[28] Quigley CA, Lederman JR (2004). Possible gatifloxacin-induced seizure. *Annals of Pharmacotherapy*.

[29] Sire S, Staub-Schmidt T, Ragnaud JM, Christmann D, Aubertin J (1996). Convulsion induced by ofloxacin. *Revue de Medecine Interne*.

[30] Traeger SM, Bonfiglio MF, Wilson JA, Martin BR, Nackes NA (1995). Seizures associated with ofloxacin therapy. *Clinical Infectious Diseases*.

[31] Walton GD, Hon JK, Mulpur TG (1997). Ofloxacin-induced seizure. *Annals of Pharmacotherapy*.

[32] Yasuda H, Yoshida A, Masuda Y, Fukayama M, Kita Y, Inamatsu T (1999). Levofloxacin-induced neurological adverse effects such as convulsion, involuntary movement (tremor, myoclonus and chorea like), visual hallucination in two elderly patients. *Nippon Ronen Igakkai Zasshi*.

[33] Simpson KJ, Brodie MJ (1985). Convulsions related to enoxacin. *The Lancet*.

[34] Fraser AG, Harrower ADB (1977). Convulsions and hyperglycaemia associated with nalidixic acid. *British Medical Journal*.

[35] Jo M, Tachi N, Shinoda M (1979). Convulsions from excessive dosage of nalidixic acid: a case report. *Brain and Development*.

[36] Rubinstein E (2001). History of quinolones and their side effects. *Chemotherapy*.

[37] Schmuck G, Schurmann A, Schluter G (1998). Determination of the excitatory potencies of fluoroquinolones in the central nervous system by an in vitro model. *Antimicrobial Agents and Chemotherapy*.

[38] Williams PD, Helton DR (1991). The proconvulsive activity of quinolone antibiotics in an animal model. *Toxicology Letters*.

[39] Akaike N, Shirasaki T, Yakushiji T (1991). Quinolones and fenbufen interact with GABA(A) receptor in dissociated hippocampal cells of rat. *Journal of Neurophysiology*.

[40] Brochot C, Marchand S, Couet W, Gelman A, Bois FY (2004). Extension of the isobolographic approach to interactions studies between more than two drugs: illustration with the convulsant interaction between pefloxacin, norfloxacin, and theophylline in rats. *Journal of Pharmaceutical Sciences*.

[41] Yoshino T, Noguchi M, Okutsu H, Kimoto A, Sasamata M, Miyata K (2005). Celecoxib does not induce convulsions nor does it affect GABAA receptor binding activity in the presence of new quinolones in mice. *European Journal of Pharmacology*.

[42] Poe TE, Marion GS, Jackson DS (1984). Seizures due to nalidixic acid therapy. *Southern Medical Journal*.

